# Modular health services: a single case study approach to the applicability of modularity to residential mental healthcare

**DOI:** 10.1186/1472-6963-14-210

**Published:** 2014-05-09

**Authors:** Rutger Soffers, Bert Meijboom, Jos van Zaanen, Christina van der Feltz-Cornelis

**Affiliations:** 1 Department of Management, Tilburg University, Warandelaan 2, Tilburg 5037 AB, Netherlands; 2 Department of Tranzo, Tilburg University, Warandelaan 2, Tilburg 5037 AB, Netherlands; 3 GGz Breburg, Tilburg, Lage Witsiebaan 4, Tilburg 5042 DA, Netherlands

**Keywords:** Mental healthcare, Residential care, Modular healthcare, Service modularity, Healthcare modularity, Assisted living facilities, Chronic psychiatric patients

## Abstract

**Background:**

The Dutch mental healthcare sector has to decrease costs by reducing intramural capacity with one third by 2020 and treating more patients in outpatient care. This transition necessitates enabling patients to become as self-supporting as possible, by customising the residential care they receive to their needs for self-development. Theoretically, modularity might help mental healthcare institutions with this. Modularity entails the decomposition of a healthcare service in parts that can be mixed-and-matched in a variety of ways, and combined form a functional whole. It brings about easier and better configuration, increased transparency and more variety without increasing costs. Aim: this study aims to explore the applicability of the modularity concept to the residential care provided in Assisted Living Facilities (ALFs) of Dutch mental healthcare institutions.

**Methods:**

A single case study is carried out at the centre for psychosis in Etten-Leur, part of the GGz Breburg IMPACT care group. The design enables in-depth analysis of a case in a specific context. This is considered appropriate since theory concerning healthcare modularity is in an early stage of development. The present study can be considered a pilot case. Data were gathered by means of interviews, observations and documentary analysis.

**Results:**

At the centre for psychosis, the majority of the residential care can be decomposed in modules, which can be grouped in service bundles and sub-bundles; the service customisation process is sufficiently fit to apply modular thinking; and interfaces for most of the categories are present. Hence, the prerequisites for modular residential care offerings are already largely fulfilled. For not yet fulfilled aspects of these prerequisites, remedies are available.

**Conclusion:**

The modularity concept seems applicable to the residential care offered by the ALF of the mental healthcare institution under study. For a successful implementation of modularity however, some steps should be taken by the ALF, such as developing a catalogue of modules and a method for the personnel to work with this catalogue in application of the modules. Whether implementation of modular residential care might facilitate the transition from intramural residential care to outpatient care should be the subject of future research.

## Background

The Dutch mental healthcare sector is sizable, with over 900,000 patients treated in 2009 [1]. Although most patients are treated in outpatient care, half of the sector’s budget is spent on intramural care [2]. A large part of the intramural capacity (approximately 40% in 2009) consists of places in Assisted Living Facilities, or ALFs [2]. Most of the places are in group accommodations with a shared living room and sanitary facilities, some are stand-alone apartments. In ALFs, patients receive treatment-related care and residential care. The latter is help with activities of daily living and coping with mental disorders [3-5]. In essence, therefore, it does not concern the cure of mental ailments but primarily assistance with living with mental ailments, such as assistance by provision of food and laundry services. In the present study, we will focus on residential care.

Currently, the specialty mental healthcare sector in the Netherlands has to limit its rising costs [6]. Cost reductions are largely to be realised by reducing intramural capacity – the number of beds – with one third by 2020 and treating more patients in outpatient care, because Dutch ALFs have substantially more beds than the international average and because these beds are costly [6-8]. This development may have to do with the fact that in the 80s and 90s of the former century, the European mental healthcare sector was characterised by deinstitutionalization of chronic psychiatric patients. This, however, had limited results in the Netherlands so far. Compared with other countries, the Dutch specialty mental healthcare sector, including residential mental healthcare, still has a high number of beds [8]. In particular, the number of places in intramural long-term care will decline. This transition from inpatient to outpatient care necessitates making patients currently in intramural long-term care as self-supporting as possible to enable them to live outside ALFs – in which case they will receive ambulatory care (as opposed to residential care). In turn, this objective implies a need to tailor the residential care patients in ALFs receive to their needs for self-development.

Theoretically, the modularity concept might help mental healthcare institutions to reorganise their care in such a way that they enable patients currently in intramural long-term care to become as self-supporting as possible. This concept originates from the Operations Management domain and is a way to (re) organise the way a product or service is offered. It concerns the decomposition of a system (e.g. a service) in parts that can be managed independently and used interchangeably [9,10]. Those parts can be mixed-and-matched in a variety of ways [11], and combined to form a functional whole [12]. Modularity might facilitate staff to encourage patients currently in intramural long-term care to become as self-supporting as possible, because it is known in other settings to bring about easier and better configuration, increased transparency and more variety [13], without increasing costs (e.g. [14-16]). Therefore, application of modularity may facilitate customisation of care towards the patients’ goals for self-support and self-fulfilment that are needed for the transition from inpatient to outpatient care.

In recent years, the application of modularity in healthcare has gained attention, as it enables cost reductions and responsiveness towards patients’ needs (e.g. [17]). However, research on healthcare modularity is still scarce and research on modularity in the mental healthcare sector is even scarcer. Two nameable contributions are by Chorpita, Daleiden and Weisz who develop a modular architecture for psychotherapeutic treatments, and by Van Brunt who decomposes validated cognitive-behavioural treatment programs into free-standing modular parts [18,19]. However, both studies focus on treatment-related care. So far, no studies could be identified that focus on modularity in residential mental healthcare. Hence, it is unknown whether it is possible to reorganise the residential care offered by ALFs using the modularity concept. The aim of this study therefore is to explore whether the concept of modularity can be applied to the residential care provided in ALFs of Dutch mental healthcare institutions. For this purpose, we will explore which prerequisites would need to be fulfilled before a mental healthcare organisation might be able to reorganise its residential care using the modularity concept. Next, we will investigate whether these prerequisites might actually be fulfilled in a specific residential care facility (an ALF) of a Dutch specialty mental healthcare institution. This way, this study aims to explore the applicability of the modularity concept to the residential care provided in Assisted Living Facilities (ALFs) of Dutch mental healthcare institutions.

### Theoretical background

A mental healthcare institution needs to fulfil certain prerequisites to be able to successfully reorganise its residential care using the modularity concept. First, it should be possible to distinguish independent, interchangeable parts in the residential care that may be combined in a variety of ways to form a functional whole, or modular package of residential care [9,10,12,17,20]. The scheme in which this is done is called *modular service architecture*[16,21]. Second, there should be mechanisms in place that ensure those parts can be both combined in a variety of ways, and form a coherent whole; these are called *interfaces*. The third prerequisite is the presence of a process in which the combination of healthcare parts is determined: the *customisation process*.

The independent parts of the residential care are called modules. They all bring about one function of the care, one service characteristic [16,22-24]. Modules can be grouped together based on commonality, for example on the similarity of their function [25]. This happens in *service bundles*[16]. These bundles can sometimes be divided in sub-bundles. An exemplary module can be ‘domestic cleaning’, which is grouped together with the module ‘getting dressed’ in a bundle ‘care’. This bundle can be broken down in, amongst others, ‘domestic care’ and ‘personal care’ [22,26].

To make a modular package form a functional whole – hence to successfully reorganise residential care using the modularity concept – interfaces should be present. These are mechanisms that enable interactions and communication in the provision of the healthcare service [12,24,27]. If we see a residential mental healthcare service as a piece of carpentry, interfaces would be the treatment plans and protocols that hold the piece together.

There are different categories of interfaces [11]. A distinction can be made between interfaces that *support variety* and interfaces that *ensure coherence*. An interface of the first type, such as a guideline, facilitates combinations and substitutions of modules to enable adaptation of the modular healthcare package to the patient’s needs (e.g. [12,27,28]). It does so by providing an aligning but not rigid structure [16,17]. The second type of interfaces makes the modules combined in a modular package form a functional whole [16,29,30]. These interfaces are fixed and rigid rules such as procedures and protocols [28,31]; an exemplary rule is that the module ‘shopping for food’ always has to be performed before the module ‘cooking food’ [13].

One can also differentiate between interfaces based on the interaction objects they concern [17]. One type of interfaces concerns the residential care modules, whereas another concerns the people involved in the residential care provision. The former type supports and directs the interactions and interdependencies between modules; an example is a treatment plan (e.g. [17,18]). Interfaces of the latter type, such as an electronic patient file, support and direct the information exchange between service providers and between service providers and patients [16,17,31]. Based on the two dimensions described above, De Blok advances a matrix of interface classifications; see Table 1.

**Table 1:** Classification of interface categories

**Interacting objects**			
		***Modules***	***People***
**Aim**	***Variety***	Substitution interfaces	Information guiding interfaces
	***Coherence***	Arrangement interfaces	Information rationalising interfaces

Source: Adapted from [17], p. 126.

To reorganise residential care from the modularity perspective, being able to distinguish modules and the presence of interfaces is not enough. Modules can be combined with each other and form a functional whole due to interfaces, but there also needs to be a process in which the right modules are chosen for each patient, so that his needs and wishes are taken care of [14]. Hence, there should be a service customisation process in which this ‘mixing and matching’ is done [9,10,16,26].

Service customisation can occur before as well as during the care provision and modification of the residential care package can take place over time [17,26]. Generally, the formation of the residential care package starts before the residential care delivery starts [26]. A generic assessment of the patient’s needs leads to a preliminary residential care package that roughly fits the patient’s requirements. When the provision of residential care starts, the package is further adapted and adjusted. After some time, the residential care package is considered finalised but possibilities for adaptations remain.

## Methods

### Research design

Three identified prerequisites need to be fulfilled before a mental healthcare organisation can reorganise its residential care using the modularity concept. This study aims to discover whether these might indeed be fulfilled in ALFs. The research is qualitative since it involves collecting and analysing non-numerical data and explorative as it aims to seek new insights [32], which is appropriate because research on healthcare modularity is scarce [33].

We performed a case study since this allows studying a phenomenon in a specific context and the research setting cannot be manipulated, and research and theory concerning healthcare modularity are at early stages of development [33-36]. We conducted a single, typical case study, which makes in-depth analysis of a case possible [36]. This research can therefore be considered a pilot study [36].

### Case selection

Because of the aim of this research, an ALF of a specialty mental healthcare institution in the Netherlands was selected. GGz Breburg is one of the 31 Dutch integrated mental healthcare institutions that serve most of the patients in the mental healthcare sector [1]. Like most of the Dutch mental healthcare institutions, it is a member of the Dutch sector organisation for mental healthcare, GGZ Nederland [37]. It is of slightly above average size and has seven care groups [1,38]. We studied one of their ALFs, the centre for psychosis, as a typical case. The centre for psychosis is part of the IMPACT care group, and the location Etten-Leur is one of the nine ALFs of the organisation [39]. In the specific ALF selected for this case study, two care teams provide residential care to approximately 90 patients with chronic psychosis, who all receive long-term care ([39]; J. van Zaanen, personal communication, August 10 2012).

### Unit of analysis

**Figure 1:**
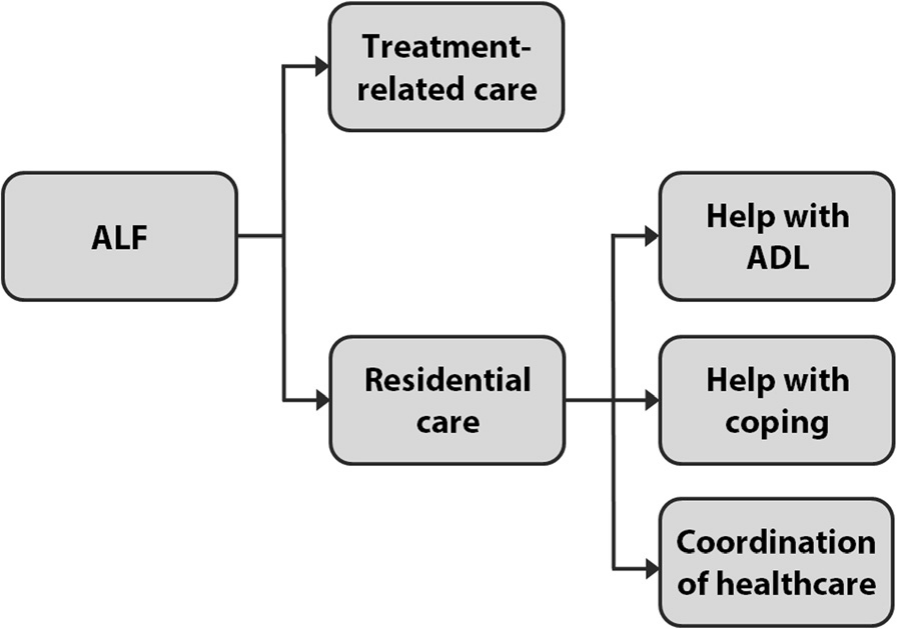
Overview of care offerings of ALFs.

As explained in the Background, ALFs offer patients treatment-related care and residential care. The latter care type is this research’ unit of analysis. It consists of help with activities of daily living (ADL), help with coping with mental ailments, and coordination of services of outside healthcare providers [3-5]. This is opposed to treatment-related care (e.g. therapies), which is generally aimed at healing or reducing the effects of a disease. Figure 1 gives an overview of the care ALFs offer.

### Data collection

This research made use of interviews, observations, and documentary analysis to collect data, and involved both primary and secondary data collection, all to allow for data triangulation [32,40,41]. Primary data were collected via interviews and observations; this provided recent data collected specifically for the current research [32]. Secondary data were collected in the form of internal and external documents to enhance the reliability of the research, because they form an unobtrusive measure not collected by the current researcher [41,42], and are of permanent nature and relatively accessible to other researchers [32].

#### Interviews

The main data collection method for this research was conducting interviews. These interviews were non-standardised because the study explores a relatively new research area [43], and because that offers a lot of flexibility during the interview, e.g. in rephrasing or replacing questions, and in probing answers [32]. The semi-structured nature of the interviews made sure important topics were addressed while leaving room to the interviewees to tell their own story [32]. On the basis of a literature review, observations and documentary analysis, a list of topics and indicative questions to be addressed in the interviews was compiled (see Table 2), so that a consistent line of inquiry was maintained throughout the interviews [36]. The face-to-face character of the interviews enabled the interviewer to clarify doubts, ensure that responses were well understood, and pick up non-verbal signals of the respondent [44]. All interviews were recorded to ensure accurate depiction of the interview [33]. Interviewees were ensured the content of the interview would remain confidential, to minimise any participant bias [32].

**Table 2:** Indicative questions for interviews

**Subject**	**Topics and indicative questions**
Service architecture	• What is residential care? What residential care does the centre for psychosis offer?
	• How is this care organised?
	• To what extent is this care standardised? Is fine-tuning for individual patients possible?
Service customisation process	• How does the assessment of the (care) needs and demands of a patient take place?
	• How is the care package composed?
	• How is the care package adapted during care provision?
Interfaces	• How is (re) configuration of services made possible?
	o Supporting interactions and interdependencies between modules;
	o Supporting information exchange between service providers and between service providers and patients.
	• How is coherence in care packages ensured?
	o Directing interactions and interdependencies between modules;
	o Directing information exchange between service providers and between service providers and patients.

Interviewees were selected using purposive, heterogeneous sampling, to make sure that persons from various functions within the centre for psychosis were selected that could provide the desired information and to touch upon all particularities of the case [32,44]. In particular, seven persons were interviewed, including residential supervisors, a care assistant, a case manager, a practical trainer for the master in advanced nursing practice and a team coordinator for day care; interviewing patients was not allowed by GGz Breburg.

While using multiple respondents increases a research’s reliability, one should not overshoot the number of interviewees as not to waste valuable resources [45]. Therefore, no more interviews were held when all interview topics had been exhaustively addressed and interviews started to produce merely known and triangulated date (i.e. redundancy was achieved) [46]. This happened after seven interviews.

#### Observations

Primary data were also collected during three and a half day of primary observations of care providers. The observations were unstructured with the observer as participant, which is appropriate because beforehand there was no definite idea of the aspects that needed focus [44]. This type of observation allowed the researchers to completely focus on the research and immediately take notes [32].

#### Documentation

Thirdly and finally, empirical data were collected by means of documentary analysis. Both internal documents (e.g. overviews of the day care provided), and external documents (e.g. publications concerning mental healthcare) were analysed. A summary of the data collection methods can be found in Table 3.

**Table 3:** Summary of data collection methods

**Type of data**	**Data collection methods**
Primary data	Interviews
	• Seven interviews
	• Semi-structured
	• Face-to-face
	• Recorded
	• Confidential
	• Interviewees from various functions within the centre for psychosis
	Observations
	• Three and a half day of observations
	• Unstructured
	• Observer as participant
Secondary data	Documentary analysis
	• Internal documents
	• External documents

#### Ethics and consent statement

The study design was approved by the scientific board of GGz Breburg. The research did not entail direct interactions with patients, but only with care providers. Because it was not aggravating to patients in any way and involved only minor patient contact, the study was exempt from requiring approval from an ethics committee. Written informed consent was obtained from the interviewees, and the manager of the centre for psychosis provided his written approval of the research.

### Data analysis

All interviews were transcribed, after which the transcripts were checked by the interviewees (i.e. member validation) to see if misunderstandings had occurred, to increase the research’s validity [33,41,47].

The gathered data were analysed using the procedure developed by Miles and Huberman (as cited in [32]). It consists of three concurrent sub-processes:

• Data reduction, where data are transformed and condensed;

• Data display, where data are displayed in a meaningful way; and

• Drawing and verifying conclusions.

The main part of the data reduction involved coding the transcripts of the interviews [33,45]. The software package ATLAS.ti 5.5 was used to improve the research’s reliability [33]. This software package was also used to manage the coded interviews, the observation memos and the collected documents. A preliminary coding scheme was based on literature; during the coding process, some codes were altered or removed, and in-vivo codes were added [32,33]. Next, the reduced data were displayed using a network and matrices (see the Results section below) that proved useful to see patterns and relationships in the collected data [32]. Finally, conclusions were drawn from those data displays [33]. The research’s findings were compared and contrasted with existing literature, and member validation was applied, to verify those findings and increase the validity of the research [33,40,41]. Table 4 summarises the tactics used to assure construct validity, external validity and reliability of the research.

**Table 4:** Tactics used to ensure the quality of the research

**Aspects of research quality**	**Tactics used in this research**
Construct validity	• Triangulation of data and data collection methods
	• Documentation of research process
	• Member validation of interview transcripts
	• Review of draft versions of the research report by a research expert and a sector expert
External validity	• Documentation of research process
	• Rich presentation of findings
	• Selected interviewees for maximum variation
Reliability	• Documentation of research process
	• Creation of case study database
	• Usage of secondary data
	• Usage of software package for coding of transcripts
	• Triangulation of data and data collection methods
	• Multiple respondents
	• Interviewees knew the interviewer
	• Confidential interviews
	• Use of face-to-face interviews
	• Member validation of interview transcripts
	• Avoiding expression of opinions by interviewer
	• Taping and verbatim transcription of interviews

## Results

Three prerequisites need to be fulfilled before a mental healthcare organisation can reorganise its residential care using the modularity concept. Below, we will present for each prerequisite to what extent the prerequisite is already fulfilled in the centre for psychosis.

### Modular service architecture

**Figure 2:**
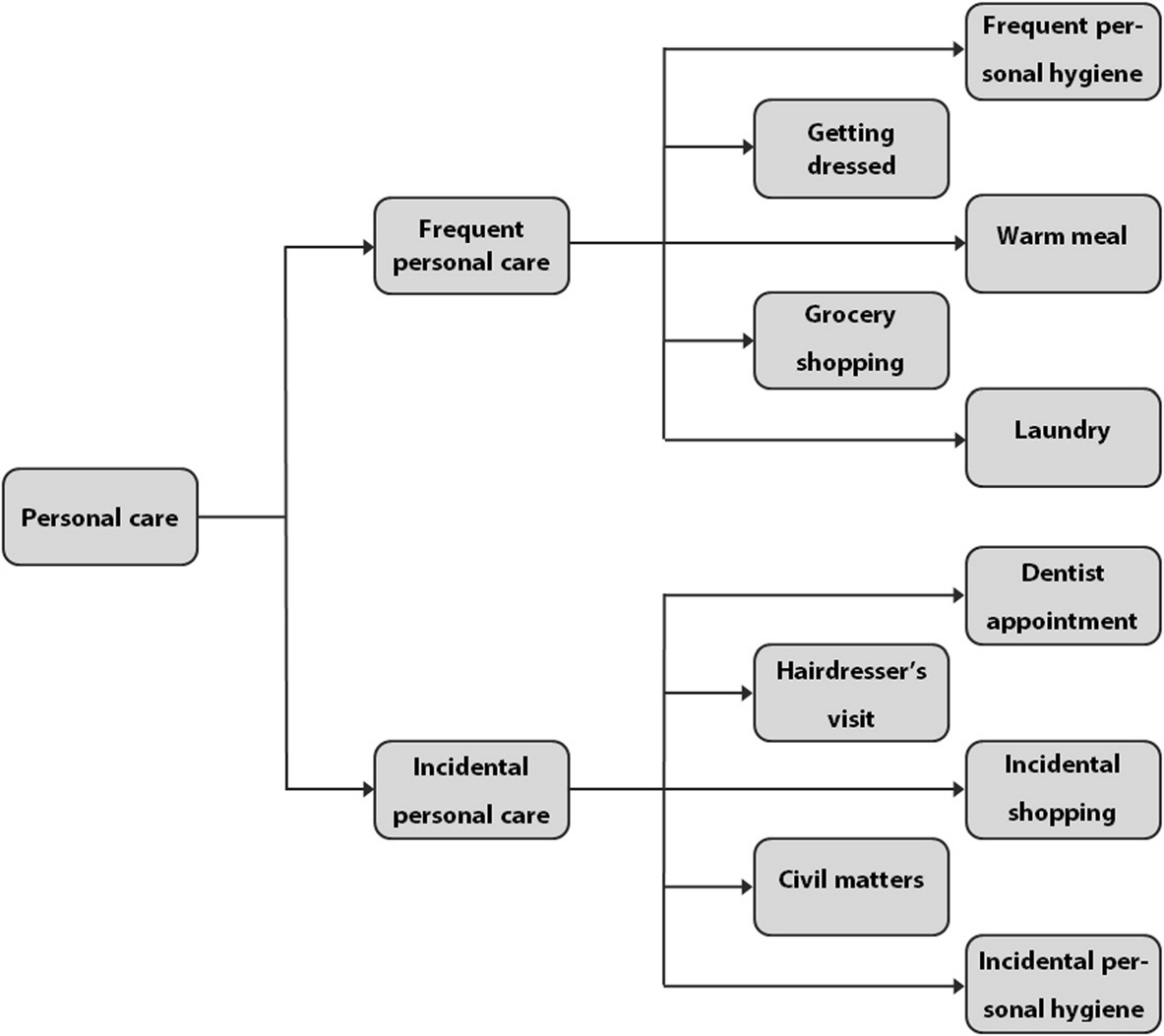
Sample of decomposition of care offered by the centre for psychosis.

As the first prerequisite for modular healthcare offerings it should be possible to distinguish modules: independent, interchangeable parts in the residential care that all bring about one function of the care. The residential care offerings of the centre for psychosis are not clearly categorised, labelled or grouped. However, based on the interviews, observations and documentation we were able to decompose a large part of the residential care in modules. In turn, these modules could be grouped in service bundles and sub-bundles based on their function. This decomposition was checked using member validation. A sample of this decomposition is depicted in Figure 2 below; a more comprehensive overview can be found in the Additional file 1. The distinguished modules come in different variants (the module ‘warm meal’ for example, comes in amongst others the variants ‘ready to eat, cold’; ‘ready to eat, warm’; and ‘patient cooks himself’) – these variants are omitted in the figure below for briefness, but are mentioned in the Additional file 1.

For a part of the residential care offerings it was not possible to identify modules. This concerns two aspects of the residential care. The first aspect is care tailored to very diverse and individual needs of patients (e.g. when they have a wish to learn something). This care is so diverse and all possible care deliverables are provided so sporadically that it is not feasible to distinguish modules. The second aspect consists of conversations with patients. These conversations are truly unstandardized so breaking them down further is impossible. Interestingly, the aim of these conversations is twofold: they are partly meant to directly help the patient (i.e. by providing a listening ear) – so that they are care deliverables in themselves –, and partly to learn about the patient’s situation – in that sense they can be seen as interfaces.

### Interfaces

The second prerequisite to reorganisation of the residential mental healthcare is the presence of interfaces; the mechanisms in place that ensure modules can be combined in a variety of ways, and are delivered as a coherent care package. Our research identified a significant number of interfaces at the centre for psychosis. Based on their aim (coherence or variety) and the interacting objects they concern (modules or people) they are grouped in Table 5.

**Table 5:** Identified interfaces

**Interacting objects**			
		***Modules***	***People***
**Aim**	***Variety***	Substitution interfaces	Information guiding interfaces
		Barely present; only overview of day care modules	• Meetings with every change of shifts
			• Care provider meetings three times/week
			• Six-weekly care team meetings
			• Six-weekly general policy meetings
			• Care package evaluation conversations
			• Regular conversations with patients
	***Coherence***	Arrangement interfaces	Information rationalising interfaces
		• Strict planning rules regarding medication and some physical screenings	• Electronic patient file
		• Agenda used for all appointments	• Residential care plan
		• Work schedule for some care modules	• Work division
			• Clear lines of communication

Only a minority of the interfaces concern modules. A few arrangement interfaces, such as planning rules for medication provision, could be identified, but there are no planning rules for a smooth flow of the rest of the care. Remarkably, no substitution interfaces were found except for an overview of the modules delivered by the day care centre. A large number of interfaces concerning people could be identified. In particular, there is a strong presence of information guiding interfaces such as care provider-patient meetings for evaluation of care packages [17]. Some delivered care serves a double function as an interface: conversations residential supervisors have with patients are not only care deliverables, but also information guiding interfaces. Furthermore, various information rationalising interfaces, such as a residential care plan, were found.

### Service customisation process

The data show that a service customisation process is present at the centre for psychosis. Moreover, this process largely follows the theory on how it should be. Firstly, a preliminary care package is formed before residential care provision starts. Next, when residential care delivery for a specific patient starts, the first six weeks of care provision are devoted to information gathering to fine-tune the residential care package – hereafter, the package is, in principle, finalised. However, possibilities for adaptation remain, for example based on the annual evaluations. Therefore, we conclude that the third prerequisite to reorganising residential mental healthcare using the modularity concept is satisfied at the centre for psychosis.

## Discussion

### Main research findings

This innovative study is the first to evaluate the Operations Management concept of modularity in a residential mental healthcare setting. This study aimed to explore whether the concept can be used to reorganise the residential care offered by ALFs of mental healthcare institutions. This reorganisation should enable the ALFs to customise the residential care that patients receive to their needs for self-development, to facilitate the transition from inpatient to outpatient care. The results show that at this particular ALF, the prerequisites for modular residential care offerings are already largely fulfilled and if they are not, they can be fulfilled.

The research has shown that modular service architecture is not yet present at the centre for psychosis. Care providers experience the residential care patients receive as a unique care package that is not composed of standard modules. However, it was possible to decompose a large part of the residential care offerings at the centre for psychosis in modules. Moreover, these modules can be organised according to their functionality. A part of the residential care could not be decomposed however, namely some very diverse and scarcely delivered care, and conversations with patients. It is striking that these conversations can be seen both as a residential care deliverable, and as an interface.

It has also become clear that at this moment, not all the interface types are represented sufficiently to allow the ALF to reorganise its residential care according to the modularity concept. However, there is no reason to conclude that this deficiency cannot be addressed. In particular, there is a lack of substitution interfaces, aiming at variety and concerning modules. Many care providers found this problematic as this caused a lack of clarity about the available care modules, which might lead to care that is less customised than desirable. The presence of arrangement interfaces, aiming at coherence and concerning modules, is also limited. Nevertheless, this does not seem to be a problem since the care providers unanimously asserted that the residential care provision runs smoothly and safely. This can be explained by the fact that interfaces in this category are particularly important for care in which mistakes are potentially disastrous, such as in medication care [17]. Indeed, medication care and some physical screenings were found to be subject to arrangement interfaces like strict planning rules. There is a strong presence of interfaces concerning people (i.e. information guiding and information rationalising interfaces), although care providers noted that usage of the information rationalising interfaces such as the electronic patient file can be improved. If substitution interfaces would be introduced and the usage of information rationalising interfaces would be improved, we think the second prerequisite to modular residential care offerings would be fulfilled.

The service customisation process is largely as it should be to enable reorganisation of the residential care using the modularity concept. A preliminary residential care package is formed before the care provision starts, and after it has started the package is fine-tuned and finalised. Moreover, the needs and wishes of the patients are taken into account in the customisation process. However, the lack of clear labelling and grouping of residential care modules and the lack of substitution interfaces comes at a cost in the care package customisation, since care providers note that the care is not individualised *enough*. Furthermore, specification of the preliminary residential care package can potentially be improved in two ways. One way is to combine modules from a pre-determined set, a menu [16,48,49]. Alternatively, the ALF can have several prototypes (i.e. combinations of modules) from which the customisation starts [16,50], for example based on different mental ailments. Besides, even though possibilities for continuous adaptation of the residential care packages exist, in practice most packages are evaluated only once a year, and if the patient is stable, no adaptations are made. In other words, care is provided without an explicit goal or aim set in terms of developing skills that would enable patients to become as self-supporting as possible and facilitate the move from inpatient to outpatient care.

All in all the service customisation process at the centre for psychosis is sufficiently apt to apply modularity; however, there is room for improvements. Some changes are indispensable, in particular in the evaluation of care packages – otherwise, reorganising the care using modularity will not help to achieve the ultimate goal, namely enabling ALFs to adapt the residential care that patients receive to their needs for self-development to make them as self-supporting as possible.

### Implications for practice

This research has made clear that to a large extent the prerequisites which would be needed to be fulfilled before an ALF might implement the modularity concept, and use it to reorganise its residential care, are already fulfilled at the centre for psychosis. Insofar as there are deficiencies in the fulfilment of these prerequisites, they can mostly be resolved. For a large part of the residential care offered by ALFs it therefore seems feasible to use modularity to reorganise the care offerings.

### Implications for research

This pilot study has provided first insights into the applicability of modularity to residential care provided by ALFs of mental healthcare institutions, and has paved the way for more research on this topic. This research provides several leads for future research:

• This research can be extended by conducting a multiple case study into the applicability of modularity to residential care provided by ALFs of mental healthcare institutions. This way, the findings with respect to this particular case can be compared with other cases.

• As an experiment, modularity could be implemented in (at least) one ALF of a mental healthcare institution. This will make it possible to test whether modularity can actually help ALFs of mental healthcare institutions to make patients currently in intramural long-term care as self-supporting as possible.

• This research finds that a part of the residential care provided by ALFs of mental healthcare institutions can be decomposed in modules, and a part cannot. Future research could quantify this finding, like Mikkola has quantified the degree of modularisation of the product offerings of two manufacturers [30].

• The results suggest that a part of the delivered residential care (i.e. conversations with patients) serves a dual purpose: it is a care deliverable in itself, and also serves as an interface. A follow-up study could address this peculiarity.

### Limitations

#### Limitations stemming from the research design

First of all, usage of a single case study design excludes the possibility for replication of the findings [36]. Although the findings could be analytically generalised, the external validity of the research is somewhat limited. Additional case studies would have enhanced the external validity and potentially sharpened the conclusions. Secondly, the research results are limited to ALFs of Dutch mental healthcare institutions. This delineation is part of the problem statement, but is a limitation of the study. Thirdly, the research explored whether the prerequisites for reorganising residential care provided by ALFs of mental healthcare institutions using modularity can be fulfilled. This is relevant because theoretically, modularity might facilitate ALFs to enable patients to become as self-supporting as possible, by customising the residential care they receive to their needs for self-development. However, this research did not actually *apply* modularity. Therefore, the advantages modularity brings for ALFs of mental healthcare institutions remain theoretical.

#### Limitations stemming from the execution of the research

Regarding the execution of the research, a first limitation can arise from the fact that the modularity ‘vocabulary’ was not known to the interviewees. This means that when analysing the transcripts, they had to be *interpreted* in modularity terms; this might have produced some perception errors. We used member validation of preliminary research results to counter these potential errors. Secondly, data triangulation using documentation was not always possible due to a lack of written records; though unlikely, this may have had some effects on the validity and reliability of this study. Thirdly, the sample of interviewees consisted of care providers only. Other stakeholders, like patients, their relatives or government officials, might have provided contrasting insights into the applicability of modularity to the residential care provided by ALFs of Dutch mental healthcare institutions.

## Conclusions

The modularity concept seems to be applicable to the residential care as it is offered by the ALF of the mental healthcare institution in the case under study. At the centre for psychosis, the majority of the residential care can be decomposed in service modules that in turn can be grouped in service bundles and sub-bundles. The service customisation process is sufficiently fit to apply modular thinking and interfaces from most of the categories are abundantly present.

For a successful implementation of modularity however, some steps should be taken by the ALFs, such as developing a catalogue of modules and a method for the personnel to work with this catalogue in application of the modules. At the centre for psychosis, the lack of some interface types can and should be resolved and care package evaluation should be improved to make implementation of modularity worthwhile. Whether such implementation of modular residential care might actually facilitate the transition from intramural residential care to outpatient care should be the subject of future research.

## Competing interests

The authors declare that they have no competing interests.

## Authors’ contributions

RS designed and carried out the study, analysed the data, and drafted and revised the manuscript. CvdF and BM conceived of the study. BM assisted in study design and data analysis, helped to draft the manuscript, and was involved in revision of the manuscript. JvZ participated in the acquisition of data, data analysis, and helped revising the manuscript. CvdF was involved in study design and revision of the manuscript. All authors read and approved the manuscript.

## Pre-publication history

The pre-publication history for this paper can be accessed here:

http://www.biomedcentral.com/1472-6963/14/210/prepub

## Supplementary Material

Additional file 1 Modular service architecture in practice. Click here for file
