# Supplementary material for: Modular health services: a single case study approach to the applicability of modularity to residential mental healthcare
Source: BMC Health Serv Res. 2014 May 9;14:210. doi: 10.1186/1472-6963-14-210 (PMC4101855; doi:10.1186/1472-6963-14-210)
Supplement: Additional file 1 — Modular service architecture in practice. [file 1472-6963-14-210-S1.docx]

# Additional file 1: Modular service architecture in practice

| **Service bundle** | **Sub-bundle** | **Module incl. variants** | **Explanation** |
| --- | --- | --- | --- |
| Living form | --- | *Living form*  Shared house on terrain  Apartment on terrain  Shared house off terrain  Household off terrain | ‘Terrain’ is the terrain of GGz Breburg in Etten-Leur, living off terrain means living in a residential area in Etten-Leur. The first three residence variants are owned by GGz Breburg. |
| Personal care | Frequent personal care | *Frequent personal hygiene*  No help  Encouragement  Help of CP  *Getting dressed*  No help  Encouragement  Help of CP  *Warm meal*  In restaurant  Ready-to-eat, cold  Ready-to-eat, warm  Housemate cooks  Patient cooks himself  With CP  (towards independence)  With CP (stabilising)  *Grocery shopping*  No help  Help of housemate  Help of CP (stabilising)  Help of CP  (towards independence)  Send list to depot  *Laundry*  No help  Encouragement  Done by CP in patient’s home  Done in laundry facility | This concerns for example showering, shaving, taking care of one’s hair, etc. Help can be mild (doing barely nothing) or strong (doing almost everything for the patient).  Help can be mild (doing barely nothing, e.g. only helping with stockings) or strong (doing almost everything for the patient).  The restaurant is owned by and on the terrain of GGz Breburg. A cold ready-to-eat meal is heated by the patient in his house. When a CP cooks together with a patient, this can be for the sake of doing so, e.g. for conversations (‘stabilising’) or as a means of teaching cooking skills (‘towards independence’).  With ‘no help’ the patient goes to the supermarket independently or orders groceries on-line. ‘Help of housemate’ generally means that the housemate does the groceries. ‘Help of CP’ can be aimed at skill building (‘towards independence’) or at stabilising (e.g. not to lose skills). The ‘depot’ is a GGz Breburg warehouse.  The laundry facility is owned by and on the terrain of GGz Breburg; laundry is collected from the patient’s house. |
|  | Incidental personal care | *Hairdresser’s visit*  No help  Encouragement  CP makes appointment  *Incidental shopping*  No help  Encouragement  Help of relatives  Help of CP (stabilising)  Help of CP  (towards independence)  *Dentist appointment*  No help  Encouragement  Accompanied by relatives  CP makes appointment  CP makes appointment, accompanied by CP  *Civil matters*  No help  Encouragement  Help of relatives  Help of CP  *Incidental personal hygiene*  No help  Encouragement  Help of CP  Specialised help | The hairdresser is located off the terrain of GGz Breburg, albeit within close distance.  Concerns e.g. shopping for clothes or accessories, hobby material, etc. ‘Help of CP’ can be aimed at skill building or at stabilising, e.g. not to lose skills. Help can be mild (making shopping list) or strong (shopping together).  The dentist is located off the terrain of GGz Breburg; this module also concerns prosthodontist appointments. It is possible that family members accompany the patient (when the CP does not do so).  Concerns e.g. requesting and collecting passports.  Concerns things like nail cutting. Specialised help is e.g. manicure and pedicure. |
| Financial care | --- | *E-banking*  Help of CP  (towards independence)  *Grocery money*  Patient manages it  Housemate manages it  Money is kept in house safe  CP manages it  *Pocket money*  Patient holds bank card  Patient gets money daily  Patient gets week money  Guardianship of relatives  *Administration*  No help  Help of relatives  Help of CP | This is a training for use of e-banking.  This money is for the entire household.  This money is for personal use by patients; some have it in their own control, others get daily or weekly portions. In many cases, patients are under guardianship of their relatives.  This concerns i.a. bookkeeping; most often, family members support and help the patient. |
| Domestic care | --- | *Cleaning*  Done by facility service  Done by patient  Help of CP  *Clearing out*  No help  Encouragement  Help of CP (stabilising)  Help of CP  (towards independence)  *Cleaning kitchen interior*  Help of CP  *Changing bed linen*  Help of CP  *Gardening*  Done by housemates  Done by patient  Done by externals | Concerns e.g. cleaning of hallway, bedrooms, sanitary facilities and kitchen exterior. The frequency of help by facility service/CP differs. Facility service only works on the terrain.  ‘Help of CP’ can be aimed at skill building or at stabilising, e.g. not to lose skills. Help can be mild or strong.  This is a part of cleaning not done by facility service. Frequency is low.  Mostly, CPs do this together with patients.  Some of the accommodations have small gardens. The externals can also be patients living in another accommodation. |
| Health-related care | Psychiatric care | *Monitoring*  Done by CP  *Scaling up*  Intensify care  Crisis intervention  *Education of thirds* | This is a part of care everyone gets.  When a patient (might) become(s) unstable, higher level CPs are involved.  Concerns educating and supporting the patient’s family and friends, to create understanding of the patient’s situation. |
|  | Medication care | *Long-lasting medication*  Collected at pre-set times  *Regular medication*  Directly from pharmacy  From CP in weekly doses  From CP in daily doses  Collected at pre-set times  Distributed at pre-set times  *If-needed medication*  Controlled by patient  Collected at pre-set times | Called ‘depot’, this medication (usually) lasts several weeks.  Only people living off the terrain receive the medication from the pharmacy. Daily and weekly doses are always collected at the CPs office at pre-set times. Collecting/distributing happens at three pre-set times during a day.  Medication for when a patient feels anxious or otherwise thinks some more medication is needed. |
|  | Somatic care | *Physical monitoring*  According to protocol  Metabolic screening  Screening for visit somatic doctor  Other  *Visit to somatic doctor*  CP makes appointment  CP makes appointment, accompanied by patient  CP makes appointment, arranges family goes with patient  *Hospital visit*  CP makes appointment  CP makes appointment, accompanied by patient  CP makes appointment, arranges family goes with patient  *Physical care*  Varies | Some physical check-ups are standard, like the screening patients using Leponex get. When patients will visit a somatic doctor, they have to be physically screened beforehand (e.g. on blood pressure). ‘Other’ concerns individualised screenings.  Patients living on the terrain go to a somatic doctor employed by GGz Breburg and located on the terrain; patients living off the terrain go to a general practitioner outside the terrain. A CP always makes the appointment, and often arranges family to go with the patient.  A CP always makes the appointment, and often arranges family to go with the patient.  This care varies a lot and is very much individualised. An example is wound care. |
|  | Comorbidity care | *Monitoring*  Done by CP  *Scaling up*  Intensify care  Crisis intervention | Comorbidity is the presence of a disorder or disease next to the psychiatric ailment, e.g. drug addiction. Everyone with comorbidity is monitored, when a patient (might) become(s) unstable, higher level CPs are involved. |
| Day care | Incidental activities | *Incidental activities*  Varies | These activities vary a lot. Examples are a table tennis tournament and a barbecue. |
|  | Sports | *Tai-chi*  No help  Under guidance  *Badminton*  No help  Under guidance  *Fitness*  No help  Under guidance  *Football*  No help  Under guidance  *Swimming*  Under guidance | These are practised on the terrain of GGz Breburg except for swimming. (Most) often practised under guidance. |
|  | Work | *Work*  Varies | Only a few patients work; Individual Placement and Support (IPS) is on the rise. |
|  | Work-related  activities | *Administrative project*  Under guidance  *Bicycle workshop*  Under guidance  *Small farm house*  Under guidance  *Archiving in museum*  Under guidance  *Residential centre for elderly*  Under guidance  *Gardening*  No help  Under guidance  *On-terrain shop*  Under guidance | All activities are on the terrain of GGz Breburg, except for volunteering in the residential care centre for elderly Het Anbarg. The administrative project concerns e.g. making brochures for GGz Breburg. There is a small shop on the terrain of GGz Breburg were e.g. used clothes are sold. |
|  | Hobby activities | *Music lessons*  Under guidance  *Fiddling*  No help  Under guidance  *Darts*  No help  Under guidance  *Spirituality/churching*  No help  Under guidance  *Computer course*  Under guidance  *On-terrain café*  No help  Under guidance  *Billiards lessons*  Under guidance  *Strolling*  No help  Under guidance | Most hobby activities are practised under guidance. The on-terrain café is a society on the terrain of GGz Breburg where patients can sit and relax. There is a small chapel on the terrain, and a church nearby. |
